# Occupying ‘in-hospitable’ spaces: Parental/primary-caregiver perceptions of the impact of repeated hospitalisation in children under two years of age

**DOI:** 10.1371/journal.pone.0228354

**Published:** 2020-01-30

**Authors:** Karen McBride-Henry, Charissa Miller, Adrian Trenholm, Tara N. Officer

**Affiliations:** 1 School of Nursing, Midwifery, and Health Practice, Faculty of Health, Victoria University of Wellington, Wellington, New Zealand; 2 Health Services Research Centre, Faculty of Health, Victoria University of Wellington, Wellington, New Zealand; 3 Kidz First Hospital, Counties Manukau District Health Board, Auckland, New Zealand; Leiden University, NETHERLANDS

## Abstract

The experience of having a child hospitalised is stressful and disrupts families in myriad ways; however, the experiences of parents/caregivers who encounter repeated admissions of a child with acute lower respiratory infections are under-researched. This project aims to explore these experiences, from a qualitative perspective, using the philosophical tenets of reflective lifeworld research. The research included 14 face-to-face interviews with parents, grandparents, or primary caregivers, of children who, whilst under two years of age, were admitted to hospital multiple times with a lower respiratory infection diagnosis. Many of the participants were from Māori or Samoan ethnic backgrounds. The findings of this single site study revealed that these parents/caregivers' experiences were characterised by feelings of powerlessness, offering descriptions of hospitals as harsh and difficult places to reside, they are ‘in-hospitable’. The findings suggest that repeated hospitalisations created a cycle of stressful experiences that impacted both familial relationships and interactions with society. This study draws attention to this previously obscured population group, and calls health care practitioners and policy advisors to engage differently over issues involving families in similar positions.

## Introduction

New Zealand’s rate of hospital admissions for lower respiratory infection (LRI), including pneumonia and bronchiolitis, is very high compared to international standards [1]. LRI is a significant health burden for New Zealand’s paediatric population [1], particularly for those under two years of age where, nationally, 10.3% of children in this age group, are hospitalised annually with LRI [1, 2]. The relative rate of admission for LRI is 5.2 times as frequent in Pacific and 2.9 as frequent in the indigenous Māori populations, as in New Zealand European children [2]. Similarly, children in the lowest socioeconomic quintile have 4.9 times the rate of admission than children in the highest quintile, with 20% of children readmitted with repeated LRI [1]. There is growing evidence of a link between LRI in the first two years of life and recurrent respiratory conditions in childhood, potentially leading to chronic lung disease in children and adults and significantly poorer life outcomes [3, 4]; however, there is currently no research examining the impact that multiple admissions for childhood LRI have on families; this project employs a qualitative approach to examine this issue.

### The impact of acute hospitalisation of children on families

Hospitalisation of children presents both immediate and long-term challenges for children and their families. A systematic review conducted by Shudy, De Almeida (5) [5] of 115 research studies based in intensive care units explored the impact of childhood admissions for critical illness and injury on families. The authors describe how, during initial admission periods, parents and siblings were often in shock and frightened by the hospital environment. Families stressed the need for explanations from those providing care on choice of treatment provision, including developmentally-appropriate explanations for siblings [5]. Some of the studies reviewed by Shudy and colleagues [5] focused on the needs of families whilst a child is in intensive care [6–10]. In the short term, these needs included basic physiological care (water and a place to rest), emotional support, and empathetic care providers; however, long term, critical admissions negatively impacted family cohesiveness and coping. In addition, environmental stressors that arose because of these admissions included housing and financial barriers, and employment difficulties. One of the other major findings from Shudy and colleagues’ [5] review was that virtually no research has been conducted with minority or culturally-diverse populations. The authors of the current article were likewise unable to locate research conducted within the last six years that addressed these readmission issues for minority cultural groups.

Admission of a child to hospital also strains normal family routines and can lead to post-traumatic stress symptoms for children, parents, and extended family [5–7, 9, 11–17]. Parents participating in a qualitative study on the impact of a single hospital admission highlighted how they were unable to attend to normal family-related tasks, go to work or get sufficient sleep whilst their child was sick [7, 9, 13, 16, 18]. For some parents, unexpected hospital admissions are even more terrifying because they fear their child will die from their illness [9, 18, 19]; one-off hospitalisations can also cause financial disruption resulting in families being unable to meet existing commitments. A recent Australian study demonstrated the significant financial burden associated with an acute childhood hospital admission, estimating that, on average, families spend $589 Australian daily because of such events [20]. In addition, researchers demonstrate that toddlers living in families that struggle financially suffer significantly poorer health outcomes [21].

### The impact of repeat hospitalisations on children and families

The ovarian article on the issue of repeat hospitalisation remains Burke and colleagues’ [8] grounded theory study on the parental experiences of caring for chronically ill children. These researchers highlighted how parents initially believe that health care professionals (HCP) do not provide parents with all information relevant to their child’s chronic illness. This feeling is reinforced when parents observed the same treatment delivered differently at each visit. Burke, Kauffmann (8) [8] described this cycle continuing until parents ‘reluctantly take charge’ of their child’s care. During this phase, parents expressed a need to be vigilant, being always present to care for their child’s needs. At times, these parents assumed responsibility for the role that HCPs normally held and as a result advocated for their child when expected progress did not occur. The downside of this process was that parents became exhausted and needed to ‘take a break’ from caring. These findings were like those offered by Hudson and colleagues [13] who examined risk and protective factors related to the chronically ill child, the parent-child relationship, family structure, resources and the community environment. Both these studies focus on the experiences of parents with disabled or chronically ill children experiencing repeated hospitalisations.

There is currently no research addressing the impact on parents and families of situations where their child has repeated hospital admissions for acute illnesses. In addition, there is no research addressing such issues for ethnic minority populations. This project examines parental or primary caregivers’ perceptions of the impact on the wider family when having a child hospitalised for repeated LRI. In addition, this study is set in a geographic location where many minority population groups live.

### The research site

This study was set in an 82-bed children’s regional hospital that provides acute emergency and inpatient care for children 0–14 years. The hospital is part of a District Health Board (DHB) that provides health services to people in a specified geographical area in South Auckland where 119,280 of the population are between 0–14 years, this represents 13% of all New Zealand children within the same age range. The paediatric population in this region consists of a variety of ethnic groups including 29.1% Pacific, 26.4% European, 24% Māori, 18.5% Asian, and 2% designated as ‘other’ [22].

The region has high levels of socio-economic deprivation; 58% of the birth cohort lives in the most socio-economically deprived quintile of New Zealand [22, 23]. In this same population, researchers have identified that 25% of those admitted with LRI live with more than seven other people in the same house and 33% live with four or more children [24]. Results from the same study also indicate that 27% have inadequate household heating. The impact of poverty on populations is well documented; hand in hand with this issue, in New Zealand, health-related inequities disproportionately burden Māori and Pacific families, who face issues in accessing health services across the lifespan [25].

Within the catchment area of this DHB, LRI is one of the leading causes of hospital admission for children, the primary diagnosis is mostly bronchiolitis or pneumonia [24]. Children in this area have a higher LRI hospitalisation rate than the rest of New Zealand, with almost 18% of children less than two hospitalised and Māori and Pacific children carrying an unequal burden [1, 2]. This is similar to issues documented in Alaska [26]. Limited research has explored the impact of this area of high morbidity in children and available research has limited applicability to New Zealand’s specific cultural composition.

## Methods

The research process began with cultural consultation, reflecting the requirements of the study site and the importance of considering Māori and Pacific perspectives. The DHB Māori Research Review Committee evaluated and approved this study as appropriate for Māori participants. The study team included two Māori and one Samoan researchers. Their roles included reviewing the research protocols and assisting with findings analysis. Ethics approval was obtained through the Northern Y Ethics Committee (approval number NTY/10/EXP/073).

### Research methodology

This research has its philosophical foundations in hermeneutics, or reflective lifeworld research, which is concerned with the meaning that experiences have for people. This research methodology was explained extensively by nursing researchers Benner (27), Ironside (28), and Dahlberg and colleagues [27–31]. Historically, its roots are in the philosophical work of Heidegger (32), Merleau-Ponty (33) and Gadamer (34) [32–34]. Reflective lifeworld research provides a stance from which researchers can analyse narrative text, such as those derived through qualitative interviews. This stance incorporates tenets such as openness, historicity, and the hermeneutic circle, all of which have informed this research.

The concept of openness has been described as “a perspective free of unexamined assumptions” [35, p304], and is a means of examining a phenomenon as it presents itself [29, 34]. Even though in reality it is impossible for any researcher to be completely free from previously held biases, every effort is made to achieve this; the researcher constantly calls into question their prejudices, vigilantly assessing how these issues affect any interpretations [34]. “The important thing is to be aware of one’s own bias, so that the text can present itself in all its otherness and thus assert its own truth against one’s own fore-meanings” [34, p269]. The researcher aims to listen and be open to what the text is saying without any preconceived ideas [36]. It is from this place that understanding and the ‘otherness’ of the phenomenon can emerge [34, 37].

An individual is born into a place in history that affects the way they think and interpret the world around them. Their history travels with them, so they are always forming pre-understandings about the environment they are in, and from this flows interpretations. This concept has been defined as ‘historicity’. The influence that history has on understanding cannot be understated–one cannot understand or examine the future without first considering traditions and previously held values, because all future interpretations will emerge from this place or horizon [32, 34, 37]. Although it is not possible for a researcher to divorce themselves from pre-understanding and culture, it is important that their individual horizons are acknowledged [34].

Individual horizons are constantly changing and extending throughout life. When an individual is introduced to new ideas, concepts or languages, it creates new understandings extending these horizons [34]. When engaging in research with individuals from different cultures, such as in the present research, it is important that emergent knowledge be co-constructed, as language and culture inherently shape our interpretation of experiences.

The final philosophical concept underpinning this research is the hermeneutic circle. This concept emerged from the philosophical work on hermeneutics by Schleiermacher [29, 36, 38], and is based on the idea that when engaged in the act of interpreting an artefact (for example, an interview transcript), it is important to attend to both the whole and the parts of the artefact; this is crucial for interpretive accuracy. This has implications for the interpretive researcher because when developing an interpretation, they must always be mindful of the context surrounding the interpretation [29, 34]. Therefore, there is a need to travel constantly between the whole and the parts of narratives to build an understanding. This process is considered circular because understanding is never complete, and whenever a phenomenon is studied, new insights emerge. Understandings, and thus interpretations, also change as cultures and traditions evolve [34]. As Smythe (37) [37] describes “each understanding is taken back to all previous understandings, and moves forward to all new understandings. No one understanding stays static or fixed” [p94]. This means that anyone who reads an interpretation is issued a call to engage in the hermeneutic circle and re-interpret current understandings, thereby creating a circular motion of continual re-interpretation and new sense of the narratives.

Unlike traditional hermeneutic research, we have not drawn upon the philosophers as dialogical partners for the purposes of analysis. The reason for this atypical approach was a strong desire not to overlay interpretive lenses that could be culturally inappropriate and limit interpretive possibilities. It was important that Māori and Pacific ways of knowing, often overlooked in policy and practice, could emerge through the analysis process; this ensured the integrity of any interpretations. Given the participation of these ethnic minorities in the present research, cultural consultants working at the research site became part of the wider study and acted as dialogical project partners. This assisted in facilitating the study population’s access to interpretations of their life experiences; overlaying a particular philosophical lens may have undermined this possibility.

### Data collection

Semi-structured interviews were arranged to gather participant narratives (see Table 1). Interviews were conducted by three experienced child health research nurses; the nurses were all female, with one being Samoan and two of European-derived ethnicities; all were experienced with working alongside diverse populations. Prior to the interviews, the lead investigator ran training in qualitative interview techniques for these nurses.

**Table 1:** Interview guide.

	Question
1	How are you feeling about your child’s admission?
2	How do you view your child’s health?
3	How has your child being in hospital affected your family?
4	What has happened to your family since your child has been in hospital?
5	How has your role as a caregiver of your children changed during this time?
6	What would have made a difference for you and your family during this time?
7	Is there anything else that you would like to say about the impact of your child being in hospital?

Due to funding constraints not extending to translation of transcripts, all interviews were conducted in English. While all participants spoke English, in some cases, limited proficiency in expressing difficult emotions in another language could have influenced their responses. Individuals whose children had a diagnosed chronic condition(s) were excluded.

The research nurses reviewed all respiratory-related admissions in the hospital research site; they identified 22 eligible children who had more than two hospital admissions with acute LRI whilst less than two years old. The families of this treated population were purposively selected (a qualitative sampling strategy) and no other demographic characteristics were used as part of the recruitment process. Once these children were identified, the researchers approached their families and explained the research purpose, aims, and the interview process; these families were able to speak to the lived experiences of parenting a child with recurrent acute LRI and were selected because of this knowledge. Information sheets and consent forms were left with potential participants (the children’s caregivers) for their consideration. Eight families declined to participate in the research and 14 distinct families provided written consent to participate in the study. Reasons for declining, while not deliberately collected, included distress from having a child hospitalised with LRI. The authors had no predetermined sample size, and sampling ceased following identification of the currently hospitalised eligible population as these families were able to speak to the current effects of rehospitalisation.

Demographic information was obtained from participating families, including ethnicity, number of dependants, and the frequency of hospital admissions for their ill child (Table 2). Participants included biological parents (9 mothers, 3 fathers), one grandmother, and one legally appointed caregiver. Participants self-nominated their ethnicity; most participants indicated they were of mixed ethnicity. Participants primarily came from a Māori or Samoan background; other groups represented were Tongan, Niuean, or European. While participant sampling in this research did not explicitly select Māori or Pacific populations, the demographics outlined in Table 2, including around household size, to some extent support data presented earlier in this paper showing a high burden of LRI amongst these populations. The research team also gathered information about the participant’s home environments, including how many adults or children resided in the same home.

**Table 2:** Research participants.

	Ethnicity	Age	Relationship to child	Staying in hospital with child?	Child’s birth order	Siblings hospitalised (frequency)	Adults at home (n)	Children at home (less than 5 years old)
1	Māori	20–25	Caregiver	Yes	1	No	8	3 (1)
2	Māori European	26–30	Mother and father	No	1	No	3	2 (1)
3	Samoan	31–35	Mother	Yes	1	No	4	2 (2)
4	Māori Tongan	36–40	Father	Yes	2	No	5	2 (1)
5	Samoan Tongan	26–30	Mother and Father	Yes	4	No	2	4 (2)
6	Samoan	26–30	Mother	Yes	3	Yes (1)	3	3 (2)
7	Samoan Tongan	31–35	Mother	Yes	8	Yes (10+)	3	4 (2)
8	Māori Niuean	20–25	Mother	Yes	2	Yes	5	4 (2)
9	Samoan	20–25	Mother	Yes	3	Yes	1	3 (3)
10	Māori	20–25	Mother	Yes	2	No	2	3 (3)
11	Tongan	41+	Mother	Yes	7	No	2	6 (1)
12	Māori Tongan	20–25 (Mother)	Mother and grandmother	No	1	No	5	5 (4)
13	Samoan	31–35	Mother	Yes	4	Yes (3)	7	8 (3)
14	Samoan	20–25	Mother	Yes	1	No	4	4 (2)

Interviews were conducted whilst the child was a hospital in-patient and were recorded and generally conducted one-to-one, two interviews involved more than one participant. Interviews lasted between 25 and 60 minutes, concluding when the participant felt they had nothing additional to add.

Following interview completion, a professional medical transcriber prepared written transcripts of the audio recordings. The lead investigator reviewed transcripts for accuracy and uploaded them to NVivo, a qualitative analysis computer software programme [39].

### Analysis of interviews

Interview analysis began with an informal focus group; this was facilitated by the lead researcher and including the three research nurses. The focus group discussed the research nurses’ experiences with recruiting and interviewing participants and potential interpretive themes arising from their own informal analysis of the interviews.

Following the focus group, two members of the research team and one of the research nurses carried out this analysis. Each interview transcript was reviewed line-by-line and, as a group, descriptive themes were developed, and portions of narratives allocated to themes. Once completed, the lead researcher read and re-read the interviews and conducted additional descriptive analysis of all transcripts.

Members of the research team familiar with the methodology participated in discussions during the interpretive analysis phase; the researchers focused on the meaning of the experience for the participants. From this phase, an analysis framework was developed and reviewed for soundness and authenticity by a qualitative expert not attached to the research team.

Following the interpretive analysis phase, one Māori and one Samoan researcher reviewed the interpretive analysis. This process involved the Māori researcher examining the interpretive analysis line-by-line before meeting with the wider team to discuss her interpretation of the analysis; she confirmed the analysis and further extended interpretations. Following this, she, and the Samoan research expert, then provided written commentary highlighting areas for further refinement. Their commentary was weaved into the interpretations presented in the findings section. This approach is in keeping with the theoretical position that all interpretations involve a melding of horizons (see research methodology section), thereby providing context for each other’s worlds.

The final aspect of the research method involved reporting research findings to participants. The final report was also provided to the DHB’s Māori Research Review Committee and higher-level results were presented to the participating hospital management board.

## Results

Across the conducted interviews, three prevalent themes emerged: *Powerless*, *An ‘in-hospitable’ space*, and *Life on hold* (Table 3). In this study, participants watched their child struggling to breathe, which left them scared and blaming themselves for their child’s poor health. In addition, they considered hospitals as ‘in-hospitable’ (harsh and unwelcoming places to reside) because they were at the mercy of HCPs and their families were forced apart, which led to unstable connections within families. The repeated nature of hospital admissions also meant that their lives seemed to be ‘on hold’ as they struggled to stay in paid employment, which brought financial burdens and housing challenges. Because of the qualitative nature of this research, it is not possible to comment on whether these experiences would hold true across all cultural backgrounds. These themes are explored in the following sections.

**Table 3:** The thematic framework.

Major themes	Powerless	An ‘in-hospitable’ space	Life on hold
**Sub-themes**	Powerless to help	At their mercy	I’m not lying
	Being scared	Disrupted connections	Financial burdens
	Taking the blame	Being separated	Our house is cold

### Powerless

All participants had children who had multiple hospitalisations for LRI, ranging from three to fourteen admissions. In addition, some families had other children also suffering recurrent LRI admissions. When their child first became unwell, participants faced a situation they had not previously experienced and had no knowledge of how to protect their child; when reflecting on this, participants emotionally recounted their distress during their child’s initial illness. The mother in the following narrative describes her experience of when her young infant first became unwell with a respiratory illness.

It freaks me out … I was feeding him one day and he just went blue purple and pale for like five minutes—he was gone, and that is every time he gets sick I just keep remembering that, so I think ‘he is going to go and then I am not going to have him no more my boy’ … When he died in my arms it was my brother and my dad, my brother pulled him off me and said “what did I do”, I said “I was just feeding him, I don’t know what happened”… he stopped breathing and my brother was trying to bang the back of his back to help him breath but it wasn’t working and so my dad grabbed him off my brother and put his mouth around his mouth and just sucked all this mucus and pus and blood up from his throat … that is when he came back and we rushed him straight here [to the hospital]… (Mother, Int 12)These experiences shaped the way parents and caregivers viewed their children and their parenting; it made them realise how powerless they were to protect their babies from the LRI. The life and death nature of their experiences disrupted their taken-for-granted ideas that they could keep their baby safe.

#### Powerless to help

The participants all spoke about how difficult it was to watch their child struggle to breathe. They were able to observe and comprehend the impact of the illness on their infants, but they acknowledged they were powerless when wanting to relieve the burden of respiratory illness.

*I feel really bad and I only wish that I can carry his pain for him and he can be well* … *When I see him on oxygen I am like ah no, I feel really bad, I feel sorry for him* … *If only he can tell me what is bothering him then maybe I can, but he is not talking so I have to bring him to the professionals who know what is wrong with [him]. (Mother, Int 6)*

The impact of watching their child struggle to breathe and the powerlessness they felt introduced fear into the parenting experience. Ultimately, participants spoke of the fear that they would lose their children; this meant they lived being scared.

Speaking to the concept of powerlessness and being unable to help, another mother stated:

*I was devastated* … *I was really worried*… *so much mixed feelings… it was just really heart-breaking just watching her go through everything like the tubes and everything, all the late nights and the oxygen especially her breathing that I was more concerned about [that]* … *I just noticed the coloration of her skin and the wheezing, the vomiting… I was more concerned (Mother, Int 10)*

#### Being scared

These early experiences with their child’s respiratory illness left participants feeling powerless because they no longer felt secure that their child would survive; they lived in fear over being unable to prevent their child becoming unwell. Being scared commonly affected families, as another mother describes:

*I feel like I am walking on egg shells most of the time when I am with him*, *constantly with him I am worried… is he going to get sick its I am always walking on egg shells. (Mother, Int. 12)*

This meant their confidence in keeping their children safe eroded and their sense of agency diminished.

*I am just scared I might lose my kids ‘cause they are quite young … I am just scared because I am a single mum and there are three* … *when I look at them and it is kind of hard for them to breath it just scares me a lot too … it is hard for me because I can’t fix them, it is hard … I am scared and at the other point, I am happy that I am here [in hospital]. (Mother, Int. 9)*

*After the experience of seeing one of her children unwell with a respiratory illness, this mother was concerned she would lose all her children to illness; however, she found being in hospital reduced her fear, as she believed her child was safer in the care of HCPs*. *This sentiment was repeatedly echoed by parents and caregivers who participated in the study leading to the belief that HCPs were better at caring for their child’s health, which meant many began to blame themselves for their child’s illness*.

#### Taking the blame

Participants in this study consistently blamed themselves for their child’s illness. The lack of agency they had over their child’s wellbeing meant they analysed their parenting behaviour and attributed illness to taking their child outside, and their inability to pay for adequate housing, heat, or appropriate clothing. In addition, to taking the blame, other family members also accused them of failing to be adequate parents, which added to the emotional burden of having a frequently ill child.

*I say it is because of me, once she starts getting sick, I think what day did I not dress her up properly [for] where were we going* … *She [my mother] gets angry ‘cause she hates seeing her come in and out of the hospital … They just think it is the way I dress her* … *Sometimes I think they are right* … *[I feel] useless* … *and when I am in hospital like and she is starting to get better like I always promise myself that I won’t take her anywhere, but then when I see her getting better again I start taking her out. (Mother, Int.14)*

*Participants’ feelings of powerlessness and failure in maintaining their child’s health permeated discussions*. *Accepting the blame for their child’s illness was a way for participants to make sense of why their child became ill*. *They described that if they could unravel the reasons for the illness, then they could attempt to address them, and prevent their reoccurrence, which frequently left them needing hospital support*.

### An ‘in-hospitable’ space

All parents and caregivers participating in this study had children who had multiple hospital admissions presenting with severe respiratory illness. They all had stories of their experiences with the hospital system and its personnel. They spoke at length about the impact their experiences had on how they understood the health care system. They recognised that repeated hospital admissions significantly influenced their family life, relationships with other family members, and complicated paid employment, finances, and housing arrangements. They spoke of the challenges of having little control over a foreign environment and being at the mercy of HCP skills and attitudes. The hospital was an ‘in-hospitable’ space.

#### At their mercy

When a child was sick enough to present to hospital, parents quickly realised that things did not always progress smoothly. Families would present at the emergency department and expect their child to receive necessary assistance, including prompt diagnosis and treatment. The following narratives offered by one set of parents describe a situation where they arrived in hospital and did not receive the care they knew their child needed; this meant their daughter became more acutely unwell.

*Mother: I was actually getting stressed because the emergency department, I felt like the help was not really there … and we were taking it out on each other*. *Father: It is quite confusing … with our girl, I know when she is in distress, [but] you don’t [know] if you are really seeing it right so you want to get a doctor’s perspective on it, but then when they say that she is not [in distress]* … *What’s going on, you are a doctor, you [spent] a hundred grand studying [and] like five or ten years [studying] and you can’t even tell when a kid is sick. (Mother, Father, Int. 2)*

In this narrative, the parents had watched their child over time; they were able to recognise that she was sick and needed help. They spoke of trying to persuade each other to seek the assistance they knew their daughter needed, but they did not believe HCPs would respond and so felt powerless to help their daughter. They struggled to reconcile their parental knowledge of their child and their ability to recognise the seriousness of her illness, against the lack of HCP response to an obviously ill child. In addition to not receiving the expected quality of care, some parents experienced a lack of HCP consideration for how their child’s illnesses affected their own livelihood.

*I need help I call them [the nurse] and they take too long to respond* … *that is when I really get angry and frustrated … Getting that extra help to help me look after her really*… *I was really exhausted and tired and I walked into the office and I asked if I could turn the TV on, and the nurse looked at me and she just started laughing*. *I just looked at her and I told her “I don’t find that really funny you know”. Maybe it is a pretty silly question to ask someone to turn the TV… but she just kind of made me feel really down and out, so I just went into the room and poured my eyes out again … at the end of the day it is like people don’t really understand what I was going through at that time … and you try and explain it to them but… They are not hearing me. (Mother, Int. 10)*

The lack of care or concern displayed by HCPs at a time when parents are in an ‘in-hospitable’ space was an issue for many participants. They spoke about a general lack of understanding of how their child’s recurrent illness affected them on a moment-by-moment basis, which resulted in feelings of isolation and a sense of being completely unsupported. Participants’ relationships with the health system were punctuated with examples of misunderstanding and a general perceived lack of support. Relationships with partners and family members were also challenged by repeated hospital admissions.

#### Disrupted connections

Study participants spoke of how the repeated hospitalisation of their children often had a negative impact on their familial and community connections, in particular, the connection between partners. The frequency with which participants needed to request assistance to care for their other children and being unable to fulfil their typical role within a family, often caused friction. The relationship most often strained was the one between partners. This often occurred because of disruptions to normal family life during an admission but further continued into life outside of hospital as parents focused on maintaining their child’s health and preventing further illness. The following narrative describes a situation where a mother’s desire to keep her child well, meant she was willing to risk her relationship with her partner.

*I told him [my partner] if it is any worse, I am going to take him [our son] with me, and go to my family until he stops smoking and he was going “you threatening me?” I am not, but I am looking out for his [my son’s] health*. *“You know if you not stop smoking I am going” … Because he keeps smoking and it does not matter if he smokes outside but he comes and it smells on his clothes and hands*. *(Mother, Int. 3)*

Another example of disrupted connections was brought forth in discussions with a mother, who was separated from her children’s father, she stated:

…*then they go back to the dad [as I am in hospital with the sick child] ‘cause the dad kind of spoils them now, so is kind of hard for me now ‘cause my relationship with my kids is not the same*… *‘Cause I keep coming to hospital a couple of times and sleep over here overnight and stuff… It kind of just makes me angry…, I brought up the kids by myself and now their dad comes and all of a sudden they want to go and see their dad. (Mother, Int. 9)*

Prior to parenting a child with repeated respiratory illness, participants were concerned with routine parenting issues; however, having an unwell child meant they focused increasingly on adapting their parenting behaviours to keep their child well, potentially at the expense of other significant relationships. The call to care about their child drowned out relationships that may have been important in different circumstances. Despite these challenges, maintaining a relationship with their other children, who were not affected by illness, was also a significant source of concern for the participants and at the forefront of their minds; being separated from these children whilst in hospital with their ill child was one of the most difficult parts of the experience.

#### Being separated

Over half the participants had more than one child; they all struggled to leave their other children at home when hospitalisation occurred. Separation disrupted normal family functioning; parents were very aware that the children left at home struggled emotionally with being apart. The frequency with which these families were separated further aggravated an already difficult situation. One mother, who was a parent to eight children, spoke about the turmoil she experienced at leaving her other children at home.

*They get very angry ‘cause every time they know I am in [hospital] they are like “aagg she is always with her!”, and it makes me feel like they must feel that my attention is all to her but it is not like that … I am really close with my boys and my girls but they feel sorry for her that she is in hospital*, *but they also feel like … she is taking me away from them, but it is definitely not like that, it is certain stages, like now I have got to be here for her*. *I try and spend as much time as I have with them all. (Mother, Int. 7)*

The frequency and length of time that these families were separated significantly disrupted routines, relationships, and overall family life. The children at home felt abandoned by their parents, and the parents felt despair about the impact on their children.

*It is sad but I just keep telling her [my other daughter] that you know that baby is really sick and I try to speak to another doctor if my kids can come stay with me [in hospital] but they said they can’t* … *My kids they always sleep with me, they sleep with me and when they wake up and they can’t find me they run into the other room, so it is hard. (Int. 9)*

The experience also left them powerless to respond to their healthy child’s needs because, despite many of them pleading with HCPs to let other siblings stay, hospital policies dictated that they could not have other children staying with them. For solo parents this had massive implications as often it meant they were forced to choose between being in hospital and being with their other children, especially if they did not have anyone else to call upon for help. This further emphasised the ‘in-hospitable’ space that participants resided in and led them to put their lives on hold.

### Life on hold

Caregivers in this study frequently discussed issues of employment, financial stresses, and housing that eventuated following their child’s multiple hospitalisations. When discussing these issues, caregivers often highlighted the long-term repercussions these hospitalisations had even after their child was discharged, leaving them with a sense of life being on hold.

#### I’m not lying

All participants spoke about the impact their child’s hospitalisations had on employment arrangements. Even if one partner was not employed, and available to be in hospital without employment-related restrictions, there were still challenges with arranging time away from work to juggle family commitments during these hospitalisations for the other partner. One father spoke of how his employer had understood his situation, but he was still aware that due to the frequency of the hospital admissions he was in a vulnerable position.

*[My boss has been] not too bad so far* … *They have usually been good so far … They ask questions sometimes … Just making sure I am not lying, yeah. (Father, Int. 4)*

In addition to employers questioning their integrity, a number of participants became unemployed because of the frequency of their child’s admissions. In the following narrative, a mother, previously employed in a temporary position, describes how due to her child’s illness, her employer came to view her as unreliable and terminated her employment.

*I had to finish work, ‘cause the last time she was in [the hospital]* … *Last year I was on call work … I would be there for about three, four months but in that, in that amount of time she would be in hospital like two times, and they just got sick of the excuses, thinking I was using that as an excuse. The last time I came in [to hospital] I rang up and I asked the agency if they could get somebody to cover me because I was in hospital, she rang me back later on in that week and said that they no longer need my assistance* … *I always took her in [the hospital] discharge papers just to prove … I think it just happened too many times* … *And now I am home with family assistance … I love working … It stresses me out… (Mother, Int. 7)*

The loss of this mother’s employment had a significant impact on her family’s finances and on her sense of identity. It meant she was even more dependent on government assistance to raise her children. Her child’s illness resulted in a loss of independence; her life was on hold in relation to employment opportunities.

#### Financial burdens

Families involved in this study struggled with significant financial burdens because of their child’s repeated admissions. One participant described this as being a cycle where hospitalisation resulted in changing financial circumstances:

*‘Cause it is like a when he [my son] comes in here [to hospital] that is when the financial side goes down with us*. *(Mother, Int. 12)*

Such financial burdens resulted in families taking extreme measures to purchase necessities for life including food and clothing for their children. Often the only option for families was to approach finance companies that specialise in high-risk loans; this caused other long-term issues. One mother spoke of the impact that the repeated hospitalisations had on her.

*The kids’ food, I was already in a bad financial [state] when my partner left me … It is hard for me especially their [the children's] clothes and food … I have to ring all the finance [companies] I am dealing with to see if I can do it [pay them] the next day or the next week but I am in hospital*. *But the thing is they need all the documents from the hospital to show I was in hospital so they are not going to give me the arrears … It is kind of hard for them to understand because every time they ring me and I say I am in the hospital I don’t know if they are believing me … only when I fax the papers over like my son discharged and they know what is happening … I just catch the bus and go to the library [to fax papers] … oh well, if you love your kids you can do everything for them. (Mother, Int. 9)*

In South Auckland, where this research was undertaken, families often approach finance companies to assist in paying for staple items. For participants in this study, however, such actions coupled with repeated hospitalisations meant that they were at risk of incurring even more debt. For the mother in the narrative above, she was unable to make required payments because she could not physically go and pay her bills. This meant her integrity and willingness to pay were questioned, further victimising her during extremely difficult periods. The financial struggles these families faced also had a significant impact on the environments in which they lived.

#### Our house is cold

Living in large extended families was usual for participants (see Table 2). They spoke of various living arrangements including houses, sleep outs, and garages. They spoke of sometimes having more than one family in a single bedroom, and although it may seem unusual to others from different cultural groups, many participants considered this practice customary. These types of living arrangements also meant that families could pool their financial resources to maximise the quality of their homes; however, the participants spoke at length about how challenging their housing arrangements were in relation to keeping their children healthy because the places they lived frequently lacked insulation, were damp, and had no form of heating.

*Sometimes it is hard because there are so many kids and we don’t have enough money for clothes and stuff like that, and our house is cold so the only warmth we really got in the house is to stay in the blankets*… *Yesterday she woke up she had a cough, no runny nose or anything just a dry cough and I put her in the blanket and then within five minutes … I knew I was going to be here [in the hospital]*. *(Mother, Int. 7)*

The parents were able to articulate why poor-quality housing resulted in poorer health outcomes; however, they were unable to change the situation because of a lack of resources. One participant spoke about how she would prefer to move to her own accommodation as most of her family members smoked, which triggered respiratory problems for her son, but she was unable to afford the bond for a rental property.

*We have got my nan, my aunty and my cousin in the main house and then my uncle in the garage and then us in the back house* … *At the moment, ‘cause we do share a room with my cousin and her two kids ‘cause she has only just moved in … There is no insulation in the house itself or the roof and I would not say it is warm house … Dampness … especially around our windows… It could be impacting on his health a bit … He is always snotty, quite snotty* … *but with the cough it sort of comes and goes, he does not always have a cough… (Mother, Int. 1)*

Despite these challenging circumstances, participants made every effort to protect their children, so they could be healthy; nevertheless, life without illness was ‘on-hold’ because there were no available resources to tackle their sub-standard housing conditions.

## Discussion

The findings of this research offer unique insights into the experience of having a child who is frequently hospitalised for recurrent LRI and highlight the significant and pervasive burden that LRIs inflict on these families. The participants comprised both Indigenous and ethnic minorities and resided in an area of high deprivation. It is surprising that no previous research has explored this issue given that LRI is one of the most common cause of acute hospitalisation in children under two years old internationally and in New Zealand [1]. Although anecdotally, HCPs might be able to identify potential issues these families face, no research has explored these myriad issues inside and outside the hospital environment.

Participants provided vivid descriptions of living with an acutely and repeatedly unwell child. Parents relayed powerlessly watching their child suffer, they reported fears that their child might die, and blamed themselves for the severity of their child’s illness. Issues such as these have emerged from other researchers’ work when exploring parental experiences in the context of having a child in a Paediatric Intensive Care Unit (PICU) [18]. The parents in this study also recounted feelings of powerlessness when providing care for their child, especially in the ‘in-hospitable’ hospital context. Similarly, recent New Zealand-based research on Māori and Pacific family coping whilst caring for a child with a life-threatening chronic condition, indicates these families struggle to have their child’s needs ‘taken seriously’ within the health system [40]. Despite these insights from other researchers’ work, the authors of this current study were unable to find similar rich narratives to describe the parental/family experiences as those that emerged through this current study.

Participants described hospitals as ‘in-hospitable’ spaces, where they remain at the mercy of HCP responses to them and their children. Such feelings further disenfranchise primary caregivers, and although parents gained expertise in how to support their children whilst they were ill, they felt powerless in navigating the system because their voice was not understood. This ‘in-hospitable’ place also gives rise to the disintegration of normal family functioning, which further disrupted their connections with others. This study reflects similar findings discussed by Shudy and colleagues [5] in their earlier systematic review, which specifically explored the experiences of families when a child is admitted to a PICU.

Of interest were participants’ frustrations at being forced to separate their families during a child’s admission to hospital. Hospital policies that state only one adult can stay in hospital with a sick child caused significant hardship, especially for single parents. This further affected extended families, who were required to take time off work to care for other children who could not reside with the primary caregiver in hospital. Other researchers have emphasised the negative impact on family cohesion when a childhood hospital admission is traumatic in nature [6, 7], an issue also clearly identified by the current participants, but one not addressed in any health policy or system response.

One practical suggestion to emerge from this research is that HCPs should be empowered, perhaps through instituting clear decision tree algorithms, to make discretionary exceptions to hospital policies that limit the number of people staying with an unwell child. Such actions are important because other family members may assist in caring for a sick child, an issue also recognised by Brown [40]. HCPs, although often overworked, need to act with humanity, being mindful of the emotional vulnerability of these families, and offer sensitive and culturally-responsive care. In such situations, treatment goes beyond the immediate illness and the sick child to consider both the family and systemic issues at play. One means of facilitating more ‘hospitable’ care provision might involve wrap-around support for the child and their family. Where a child is admitted more than once to hospital with a diagnosis of LRI, it should prompt a conversation with the family about their needs with an offer to refer to appropriate support services that can further assist the family.

In the context of this research ‘in-hospitable’ spaces are places where needs are not recognised and voices of those cared for are actively silenced. It is imperative that HCPs recognise the importance of creating different ‘spaces’ in hospital, one where responsive, culturally-appropriate connections with families are possible. Gadamer created the notion of fusing-horizons to create new understanding [34]. When working alongside diverse people groups, such as the participants in this research, the ability to create spaces that allow this ‘fusion of horizons’ is challenging and complex, but it is vital to make previously in-hospitable spaces hospitable. One place to start is the inclusion of Indigenous consultants on wards and in policy development, as this creates the opportunity for culturally-appropriate and responsive spaces for these families.

All participant expressed challenges with employment, which led to financial distress, and an inability to manage housing problems because of their child’s acute LRI admissions. These are concerning findings that further underscore the ‘in-hospitable’ space in which the research cohort resides. Participants signalled that they faced many issues when attempting to integrate back into normal life, only to face another admission. The financial implications of hospital admissions for acute illness have been explored by Mumford, Baysari (20) [20]. Through their research, they were able to demonstrate that a single admission to hospital creates significant financial burdens for families, an issue further compounded for the current participants because of their cycle of repeat admissions. In addition, we also know that chronic illness leads to poorer economic outcomes for families [5], this research draws attention to the financial plight of these families suffering under multiple acute readmissions; this area warrants further exploration. Policy and funding provisions should be available for these families through support mechanisms, normally reserved for families with chronically ill or disabled children; such provisions should cease once their child returns to full health. The findings of this report further attest to the need to address systemic issues related to social determinants of health; a call also made by others [41–43].

Finally, the researchers’ choice of underpinning methodology, reflective lifeworld research, enabled the exploration of the meaning that experiences held for participants and facilitated the emergence of novel and vivid narratives. These narratives highlight the extent of the powerlessness that parents and families face in such moments. It is important that HCPs have vicarious insights, such as those offered here, into the recipients of their care so that they can be responsive and sensitive to families’ emotional needs. For the purposes of this research, the methodology, which nurses developed to explore nursing issues, demonstrated its worth for cross-disciplinary research and practice.

### Limitations and future research

Qualitative research of this nature is not intended to be generalisable to the wider population and instead presents vicarious insights into the lifeworld of participants [44, 45]. The lifeworld presented by these participants is often bleak, and perhaps influenced by factors outside the immediate control of the health system. Acknowledging potential limitations associated with using a single hospital site, this research opens the possibility of learning from the experiences of vulnerable participants and provides direction for new research in this largely unexplored area. It is recognised by the authors that the interpretation presented herein is not definitive, but instead calls the reader to engage in the hermeneutic circle and create their own interpretations of the presented themes. Future research is required to explore the impact of interventions targeted at improved housing, social support, and health navigation on this population. For example, if changes were made to current hospital policies, such as those suggested above, to accommodate vulnerable families better, would this positively affect family dynamics? Similarly, do other illnesses with a high frequency of admission in children have a comparable impact on families? Such questions are important to evaluate, particularly given issues with cultural responsiveness [43].

The present study explores the voices of minority populations, and in particular, the voice of women, who often take the role of primary caregiver. There is room, when conducting future research, to explore, in more depth, whether the experiences of parents from diverse cultural backgrounds are comparable when caring for a child with LRI. Certainly, given differences in access equity between populations, as discussed earlier, such research could prove valuable to validate further the findings of this research. Similarly, future research should also explore, in more depth, how well supported the themes presented in this research are in situations where a child’s primary caregiver is male.

## Conclusion

Caring for a sick child requiring numerous hospitalisations over the first two years of life is an extremely challenging experience for parents, leaving them and their families powerless and in ‘in-hospitable’ spaces. Parents are stressed and afraid that their child will die, but feel powerless to help, especially in the context of the hospital environment; the implications for their lives outside hospital are also substantial. This research is novel, as no one has previously explored the impact of repeat acute LRI hospitalisation on families. In addition, this is the first study to explore such issues with minority populations. It is imperative that HCPs create systems that are culturally safe and responsive to the additional needs that repeated hospitalisations have on families. Likewise, policy-makers need to be aware of the myriad issues facing these families and take steps both at local and national levels to minimise the long-term impact of repeat acute admissions.
